# The academization of midwifery: State-wide implementation of the new law governing the education of midwives (Hebammenreformgesetz) is leading to heterogeneous education

**DOI:** 10.3205/zma001330

**Published:** 2020-06-15

**Authors:** Joachim Graf, Stephan Zipfel, S. Schönhardt, D. Wallwiener, H. Abele

**Affiliations:** 1 Universitätsklinikum Tübingen, Institut für Gesundheitswissenschaften, Abteilung für Hebammenwissenschaft, Tübingen, Germany; 2 Universitätsklinikum Tübingen, Abteilung für Psychosomatische Medizin & Psychotherapie, Tübingen, Germany; 3 Universitätsklinikum Tübingen, Department für Frauengesundheit, Tübingen, Germany

## Introduction

The new version of the law regulating the education and licensure of midwives in Germany seeks to implement directive 2013/55/EU. The directive requires a 12-year secondary school education as a prerequisite for admittance to a midwifery training program [1]. The upscaling of midwifery training in Germany to university level, however, is taking place with over 70% of all entrance programmes being offered by Universities of Applied Sciences, (which offer vocational and not academic degrees) [2]. Against the recommendation of the German Council of Science and Humanities (Wissenschaftsrat), the new German regulatory law for the education of midwives allows for future educational pathways in vocational as well as academic institutions of higher learning in equal measure [3]. This trend is worrisome in so far as it may lead to an already small group of health professionals being trained in a heterogenous manner.

## University education: status quo

The demands of modern obstetrics have increased the need for midwives to be trained academically. Germany has committed itself to helping future midwives meet those demands by increasing the level of education and pre-registration qualifications necessary for licensure [4], [5]. This means in practice that the 60 vocational midwifery training programs and 10 blended programs (academic and vocational) currently offering licensure will shortly be terminated [2]. The German law regulating the training and examination of future midwives from December 2019 makes clear that midwives must be educated according to the tenets of evidence-based knowledge and practice [6]. While university medical schools offer established practice structures for midwifery students, Universities of Applied Science are reliant on entering into cooperation contracts with many different clinics [3]. Whereas University medical schools can ensure structures that are essential for a comprehensive midwifery education, such as teaching and research institutes, access to perinatal centers, simulation labs and teaching according to evidence-based practice, it is unclear how Universities of Applied Science can meet these criteria [7]. Additionally, the law has been vague on what exactly constitutes a sufficient level of training for student midwives in a system based on different contract partners with heterogenous levels of care. Due to the fact that cooperation partners with Universities of Applied Science may be arbitrarily combined, it could be conjectured that essential elements of midwifery training will be absent from their clinical practice. It was for this reason that the German Council of Science and Humanities recommended that all midwifery education take place at university medical schools [8].

## Current developments

The differences between the states in their responses to upscaling midwifery education can be seen in the following examples: Bavaria plans to offer three midwifery education programs at Universities of Applied Science [9]; in contrast, Leipzig in Saxony intends to transfer all of its former midwifery vocational training programmes to university medical schools [10]. Lower Saxony plans to admit 185 new students to midwifery programmes at four locations, three of which are university medical schools (Medizinische Hochschule Hannover, Universität Göttingen and Universität Oldenburg) [11]. Many other states have not made their intentions clear. Baden-Württemberg was the first federal state to offer both a Bachelor degree (B.Sc.) and state licensure at the University of Tübingen Faculty of Medicine [12], [13]. The midwifery programme at the University of Tübingen Medical School blends an academic learning curriculum and clinical practice in an interprofessional setting which meets the high standards of a university hospital. Beginning with the first semester, students are required to reflect on the evidence base for their clinical practice [14]. Baden-Württemberg is planning to significantly increase the number of midwifery students in order to offset the shortage of midwives due to the academization process. Preliminary experience in the academic education of midwifery students shows that the established network of university teaching hospitals is well-placed to provide larger cohorts of midwifery students with a high-quality education in both learning and practice. University medical schools are practised in the art of effficiently managing large cohorts of students, such as medical students, and would provide a more homogenous educational experience for student midwives. Against this background, state-led initiatives to establish academic opportunites for midwives after the cessation of all vocational training programs should be assessed critically [2], [3]. 

## Appeal to Germany’s medical faculties

Tübingen University has had a postive experience integrating midwifery students into their medical faculty and appeals to medical schools throughout Germany to also establish academic pathways for the study of midwifery science at their institutions. Both doctors and midwives profit from interprofessional education, preparing them for working together more effectively in the future [8]. The academization of midwifery has the potential to foster new areas of conflict around cooperation and realms of responsibility between midwives and doctors and interprofessional practice within an academic setting could work to sensitize all obstetric caregivers to the need for postive conflict resolution in the interest of the women they serve. It is particularly important for midwives who work autonomously to be educated within a framework of competencies that are grounded in evidence-based practice. 

Midwives work daily in tandem with doctors in clinical practice: they must be able to use an evidence-based framework to diagnose between physiology and pathology and to seek individual interprofessional solutions when they are needed. If the education of midwives takes place at university medical faculties, both student doctors and student midwives stand to gain. All aspiring maternal health care professionals profit from an education founded on the concepts of evidence-based medicine, particularly as many aspects of German perinatal medicine and obstetrics have yet to be fully grounded in evidence-based criteria.

The importance of establishing departments of midwifery science at medical schools helps to define the path forward for future midwives from vocational training to academization. University medical faculties offer the means and structure to facilitate research at the crossroads between clinical obstetrics, perinatology, women’s health and public health.

In Baden-Württemberg, Tübingen and its network of affiliated teaching hospitals would be willing to assist other medical faculties in the development of departments of midwifery science in order to achieve the high level of excellence that the profession deserves.

## Competing interests

The authors declare that they have no competing interests. 

## References

[1] Europäisches Parlament (2013). Richtlinie 2013/55/EU des Europäischen Parlaments und des Rates vom 20. November 2013 zur Änderung der Richtlinie 2005/36/EG über die Anerkennung von Berufsqualifikationen und der Verordnung (EU) Nr. 1024/2012 über die Verwaltungszusammenarbeit mit Hilfe des Binnenmarkt-Informationssystems ("IMI-Verordnung"). Amtsbl Eur Union.

[2] Plappert CF, Graf J, Simoes E, Schönhardt S, Abele H (2019). The Academization of Midwifery in the Context of the Amendment of the German Midwifery Law: Current Developments and Challenges. Geburtsh Frauenheilk.

[3] Bundesministerium für Gesundheit (2019). Gesetz zur Reform der Hebammenausbildung und zur Änderung des Fünften Buches Sozialgesetzbuch (Hebammenreformgesetz - HebRefG). Bundesgesetzbl.

[4] Fleming V, Holmes A (2005). Basic nursing and midwifery education programmes in Europe. A report to the World Health Organization Regional Office for Europe.

[5] Graf J, Brucker SY, Wallwiener D, Wosnik A, Zipfel S, Simoes E (2018). Akademisierung der Hebammenausbildung: Erweiterung der Kompetenzen als Beitrag zur interdisziplinären Versorgung bei Brustkrebs in der Schwangerschaft. Geburtsh Frauenheilk.

[6] Bundesministerium für Gesundheit (2019). Referentenentwurf der Studien- und Prüfungsverordnung für Hebammen (HebStPrV).

[7] Plappert CF, Graf J, Schönhardt S, Abele H (2019). Wohin führt der Weg? Die Akademisierung des Hebammenberufs im Kontext der Reformierung des Hebammenausbildungsgesetzes. Frauenarzt.

[8] Wissenschaftsrat (2012). Empfehlungen zu hochschulischen Qualifikationen für das Gesundheitswesen.

[9] Deutsches Ärzteblatt (2018). Hebammenstudiengang an drei Hochschulen in Bayern. Dtsch Ärztebl.

[10] Meine B (2019). Leipziger Uniklinik bereitet Studiengang für Hebammen vor. Leipz Volkszeitung. Leipz Volkszeitung.

[11] Niedersächsisches Ministerium für Wissenschaft und Kultur (2019). Hebammen sollen künftig studieren. Thümler: "185 Anfängerplätze zur bestmöglichen Vorbereitung auf den Beruf".

[12] Abele H, Graf J, Simoes E, Schönhardt S, Plappert CF (2919). Der Interprofessionalität verpflichtet: primärqualifizierender Bachelor-Studiengang Hebammenwissenschaft an der Medizinischen Fakultät Tübingen - ein Modell. Gynäkologe.

[13] Ministerium für Wissenschaft, Forschung und Kunst Baden-Württemberg (2018). Bundesweit einzigartig: Neuer Studiengang Hebammenwissenschaft eröffnet.

[14] Plappert C, Schönhardt S, Weinmann S, Abele H, Graf J (2019). Längsschnitt-orientierte Vermittlung von wissenschafts-basierten Kompetenzen im primärqualifizierenden Studiengang Hebammenwissenschaft.

